# Proteo‐metabolomic insights for early dual physical and cognitive impairments: A search for biomarkers of healthy aging based on muscle–brain crosstalk

**DOI:** 10.1111/acel.14407

**Published:** 2024-11-08

**Authors:** Yi‐Long Huang, Wei‐Ju Chang, Chen‐Hua Huang, Chao‐Hsiung Lin, Li‐Ning Peng, Chih‐Ping Chung, Liang‐Kung Chen, Wei‐Ju Lee

**Affiliations:** ^1^ Center for Healthy Longevity and Aging Sciences National Yang Ming Chiao Tung University Taipei Taiwan; ^2^ Department of Life Sciences and Institute of Genome Sciences National Yang Ming Chiao Tung University Taipei Taiwan; ^3^ Center for Geriatrics and Gerontology Taipei Veterans General Hospital Taipei Taiwan; ^4^ Department of Neurology Neurological Institute, Taipei Veterans General Hospital Taipei Taiwan; ^5^ Taipei Municipal Gan‐Dau Hospital (Managed by Taipei Veterans General Hospital) Taipei Taiwan; ^6^ Department of Geriatric Medicine, School of Medicine National Yang Ming Chiao Tung University Taipei Taiwan; ^7^ Department of Family Medicine Taipei Veterans General Hospital Yuanshan Branch Yilan Taiwan

**Keywords:** metabolomics, muscle–brain crosstalk, physio‐cognitive decline syndrome, plasma, proteomics

## Abstract

We employed an untargeted proteo‐metabolomic approach to profile circulating biomarkers in plasma samples from the I‐Lan Longitudinal Aging Study, aiming to identify biomarkers and pathways associated with physio‐cognitive decline syndrome (PCDS). In 115 propensity score‐matched PCDS case–control pairs, pathway analyses implicated dysregulation of fatty acid metabolism and inflammation in PCDS pathogenesis. Sex‐specific associations were observed, with disruptions in central carbon metabolism (elevated PKM, MDH1, and GAPDH; decreased MINPP1) and tyrosine metabolism (decreased MIF, DBH; increased thyroxine) characterizing in men. In contrast, perturbations in glutathione and phenylalanine metabolism, including increased ANPEP, GSTP1, and decreased pyroglutamic acid, were identified in women. Results suggest that dysregulated energy and redox homeostasis likely contribute to PCDS development. Notably, ANPEP, PKM, and MIF emerged as potential biomarkers, elucidating the muscle–brain crosstalk framework. Our findings provide insights into potential molecular mechanisms underlying PCDS and the muscle–brain crosstalk, marking progress toward elucidating biomarkers in the journey of healthy aging.

## Graphical abstract

The study identified dysregulated fatty acid metabolism and inflammation as contributors to physio‐cognitive decline syndrome (PCDS). Sex‐specific metabolic disruptions were noted, with altered central carbon and tyrosine metabolism more pronounced in men and glutathione pathway perturbations more evident in women. Several proteins, including ANPEP, PKM, and MIF, emerged as potential PCDS biomarkers, elucidating the muscle–brain crosstalk framework underlying this syndrome.

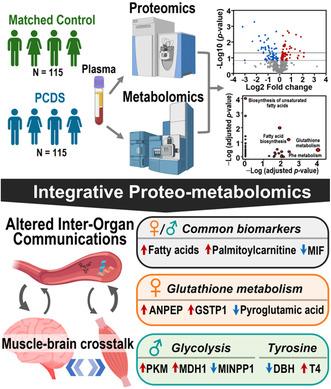

**Abbreviations**
BDT: backwards digit task
BBB: blood–brain barrier
BNT: Boston naming test
CDT: clock drawing test
CIND: cognitive impairment no dementia
CVVLT: Chinese version verbal learning test
DHEA‐S: dehydroepiandrosterone sulfate
HPT: hypothalamic‐pituitary‐thyroid
ILAS: I‐Lan Longitudinal Aging Study
LC‐MS: Liquid Chromatography‐Mass Spectrometry
MIND: mobility impairment no disability
OPLS‐DA: orthogonal partial least‐squares discriminant analysis
PCDS: physio‐cognitive decline syndrome
PSM: propensity score matching
PUFA: polyunsaturated fatty acids
QC: quality control
T4: Thyroxine
TCF: Taylor complex figure
VFT: verbal fluency test

## 1 INTRODUCTION

The concept of healthy aging has evolved significantly over recent decades, encompassing various aspects of physical, cognitive, and social well‐being in later life (Behr et al., 2023). This evolution reflects our growing understanding of the biological mechanisms underlying the aging process. In 2015, the World Health Organization issued the “World Report on Ageing and Health,” unveiling a novel paradigm of healthy aging (Beard et al., 2016). This paradigm underscores the significance of intrinsic capacity and functional ability, utilizing a life‐course approach to ensure the well‐being of older individuals over time. The intrinsic capacity is operationally defined by five elements: mobility, cognition, psychological well‐being, sensory function, and vitality, all of which are risk factors for disability and dementia in old age. While these frameworks have provided valuable perspectives, there remains a need for more precise markers of age‐related decline to inform interventions and promote healthier aging trajectories.

In this context, Physio‐cognitive Decline Syndrome (PCDS) has emerged as a promising construct for understanding and potentially intervening in the aging process. PCDS is characterized by the concurrent presence of early‐stage physical and cognitive impairments. Various forms of operational definitions have been proposed, such as cognitive frailty, and detailed comparisons between these definitions have been reviewed (Chung et al., 2021). Importantly, PCDS, as well as cognitive frailty, is not proposed as a direct marker of healthy aging itself, but rather as an indicator of accelerated aging that may signal a departure from optimal health trajectories in older adults. Recent studies have demonstrated that PCDS is associated with increased risks of disability, dementia, and mortality (Chung et al., 2021). What makes PCDS particularly significant is its potential reversibility through multidomain interventions (Liang et al., 2021), suggesting a critical window of opportunity for maintaining or restoring healthy aging trajectories. From a mechanistic perspective, there is substantial evidence supporting the potential of the muscle‐to‐brain axis as a candidate for pathophysiology, with insights drawn from a range of studies spanning cellular experiments (Yang et al., 2020), neuroimaging investigations (Lee et al., 2024), and epidemiological research (Lee et al., 2022). However, the specific molecular mechanisms linking physical and cognitive decline in PCDS remain incompletely understood. Therefore, the pursuit of biomarkers for PCDS through a more comprehensive methodology can aid in elucidating the biological mechanisms of PCDS and their pivotal implications in the context of healthy aging.

Metabolomic and proteomics profiling platforms offer potential for uncovering the molecular mechanisms underpinning PCDS, and while numerous studies have demonstrated associations between PCDS and adverse outcomes, few have pinpointed circulating biomarkers specific to PCDS. Increasingly employed techniques, metabolomics and proteomics, can quantify numerous metabolites or proteins in human biosamples, and by contrasting molecular profiles between healthy and affected groups, they can unveil distinct metabolite or protein signatures, thereby providing insights into the biological mechanisms underpinning the onset and progression of specific conditions. The application of multi‐omics methodologies presents a promising strategy to measure healthy aging (Silva et al., 2023). In this study, based on the framework of muscle‐brain crosstalk, we employed untargeted metabolomics and label‐free quantitative proteomics to systematically profile over 500 metabolites and proteins using LC–MS analysis of plasma samples from the I‐Lan Longitudinal Aging Study (ILAS). Our aim was to identify potential biomarkers and pathways associated with PCDS, thereby enhancing knowledge of PCDS pathophysiology and potentially informing new strategies for promoting healthy aging.

## 2 RESULTS

### 2.1 Characteristics of matched participants

In this study, 115 pairs of PCDS and controls, matched on the basis of propensity scores, were included. The participants' mean age was 67.0 ± 8.9 years (51–87 years) with the mean education year of 5.0 ± 4.8 years. Hypertension (42.2%) and diabetes mellitus (17.4%) were the most prevalent chronic conditions observed, with 15.2% of the cohort reporting a history of smoking and 29.1% having regular alcohol consumption, while propensity score matching was employed to ensure similarity in baseline characteristics between PCDS cases and controls (Table S1). Compared to controls, participants with PCDS exhibited worse physical and cognitive performance (*p* < 0.001, Table S1).

### 2.2 Proteomic and metabolomic profiling of PCDS

**FIGURE 1:**
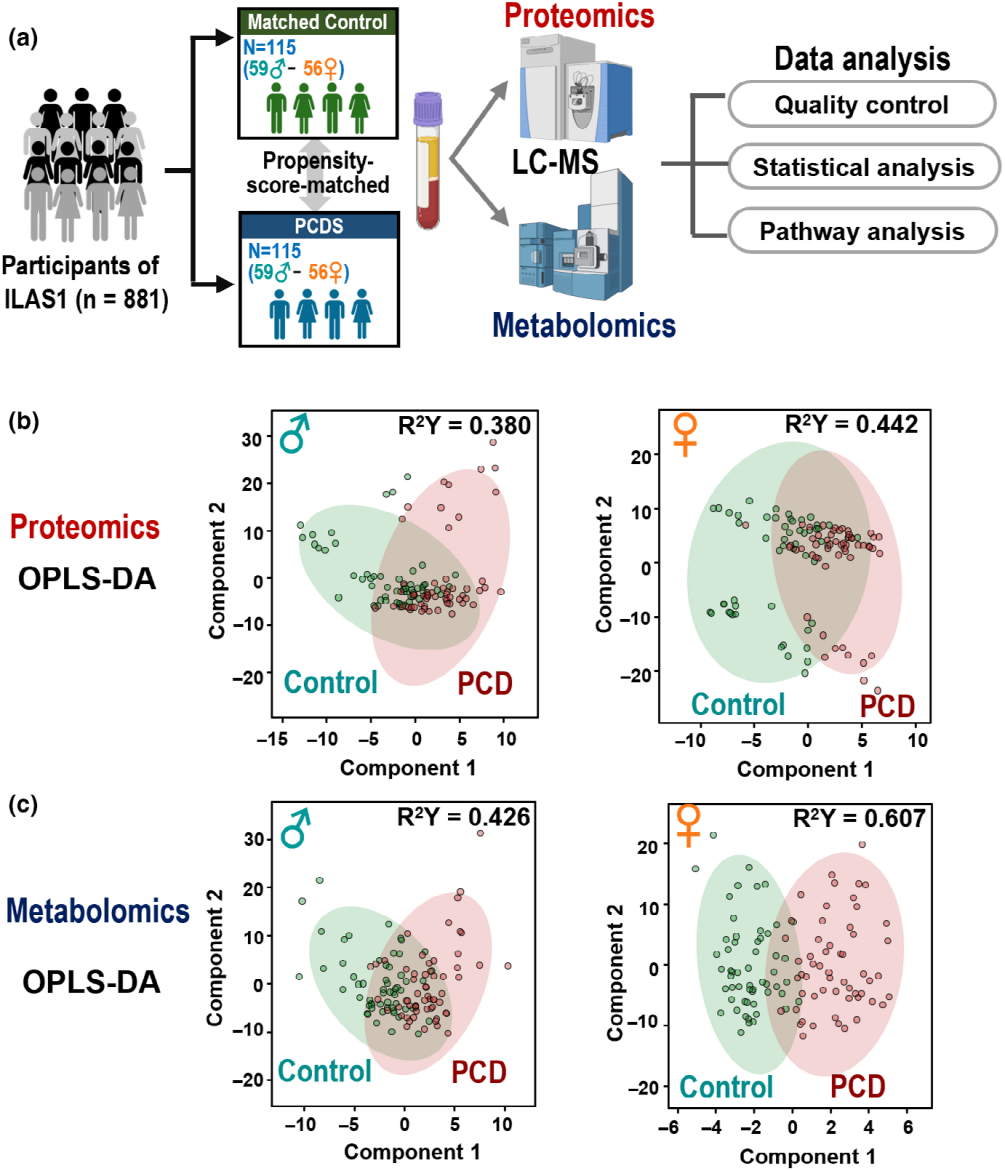
Study design and score plot of OPLS‐DA analysis for Physio‐Cognitive Decline Syndrome and robust subjects stratified by sex. (a) Study design for identification of key proteins and metabolic features derived from the LC–MS data in the propensity score–matched cohort of 115 pairs of PCDS and control. (b) OPLS‐DA model of overall proteomic profile comparing PCDS and control in females, males, and combined samples/subjects. (c) OPLS‐DA model of untargeted metabolomic profile comparing PCDS and control in females, males, and combined samples/subjects. Figure created with BioRender.com. OPLS‐DA, Orthogonal partial least‐squares discrimination analysis.

LC–MS analysis of blood plasma samples detected 516 proteomic and 532 metabolic features. For batch effect correction in the untargeted metabolomics, pooled plasma served as quality control (QC) reference samples. QC‐based normalization minimized batch effects, evidenced by stable isotope‐labeled internal standard levels across batches (Figure S1a). Normalized creatinine and DHEA‐S levels showed a strong correlation (*r* ~ 0.9) between LC–MS and biochemical blood assay measurements, indicating reliable quantification (Figure S1b). Notably, sex stratification improved group separation, especially in metabolomics where the greatest difference was observed between female PCDS and controls (R^2^Y = 0.607) (Figure 1b,c right panels; Figure S2a,b). This highlighted a sexual effect, prompting further investigation of sex‐specific protein and metabolite differences in PCDS.

### 2.3 PCDS reveals dysregulation in inflammation and fatty acid metabolism

**FIGURE 2:**
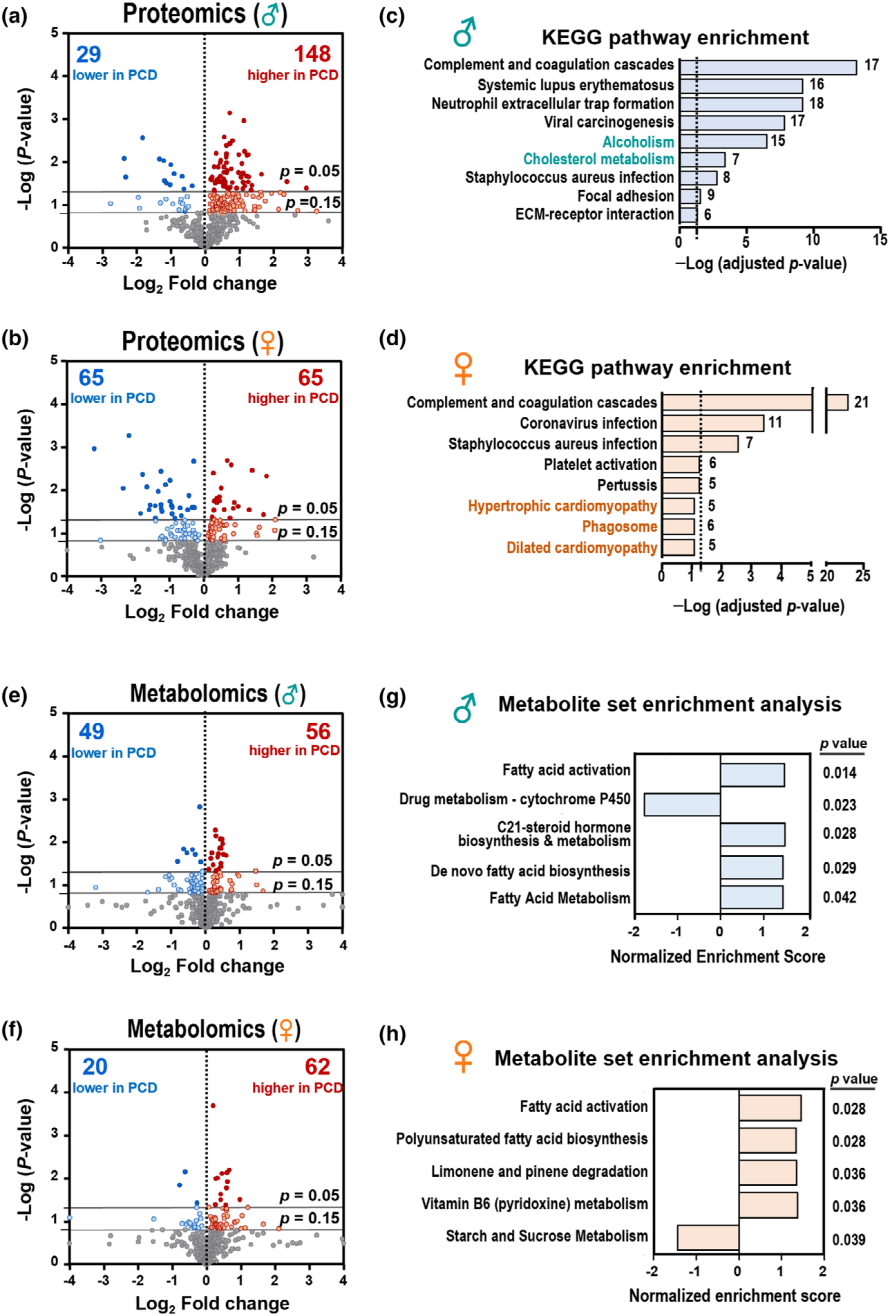
Metabolomic and proteomic profiling revealed/identified plasma biomarkers and impacted pathways of Physio‐Cognitive Decline Syndrome. (a, b) The volcano plots show the differentially regulated proteins between PCDS and control in males (a) and females (b). Two horizontal line shows the two statistical thresholds (*p*‐value of 0.05 and 0.15). Red or blue plots indicate proteins above the *p* < 0.15 threshold. (c, d) Significant KEGG pathways based on differential proteins (*p* < 0.15) of PCDS vs. Control in males (c) and females (d). The vertical dashed lines represent adjusted *p* value = 0.05. (e, f) The volcano plots show the differentially regulated metabolites between PCDS and control in males (e) and females (f). (g, h) Metabolite Set Enrichment Analysis for untargeted metabolomics of PCDS group in males (g) and females (h). Top 5 perturbed pathways (*p* < 0.05) are shown and most of the dysregulated pathways are active.

Overall, 177 and 130 differentially expressed proteins were identified in male and female PCDS participants compared to their matched controls, respectively (Figure 2a,b). Among these proteins, seven out of 177 (TIMP1, SOD1, IGHA1, CST3, S100A9, MGP, F9) and two out of 130 (GSTP1 and FBLN1) were also associated with frailty status in prior studies (Landino et al., 2021; Sathyan et al., 2020), as shown in Table S2. Pathway enrichment analysis showed that most significant KEGG pathways in both sexes were related to immune‐inflammatory, including pathways such as “complement and coagulation cascades” and infections by bacteria or viruses (Figure 2c,d). A sex‐specific observation revealed enrichment in the pathways related to alcohol (adjusted *p* = 3.21 × 10^−7^) and cholesterol metabolism (adjusted *p* = 4.13 × 10^−4^) among male individuals with PCDS. For metabolomics, 105 and 82 differentially metabolic features were found in male and female PCDS compared to same‐sex controls, respectively (Figure 2e,f). Metabolite set enrichment analysis revealed fatty acid metabolism was positively associated with PCDS in both sexes (Figure 2g,h). Additionally, cytochrome P450‐related drug metabolism was negatively correlated, and C21‐steroid hormone metabolism was positively associated with males with PCDS. For females, most PCDS‐related pathways were increased except starch and sucrose metabolism. Due to the abundance of blood coagulation and immune system proteins in the plasma, changes in metabolism‐related proteins are thought to be relatively minor. Therefore, integrated proteo‐metabolomic analysis was expected to provide a more comprehensive overview of PCDS‐related metabolic alterations.

### 2.4 Integrated proteo‐metabolomic analysis of PCDS

**FIGURE 3:**
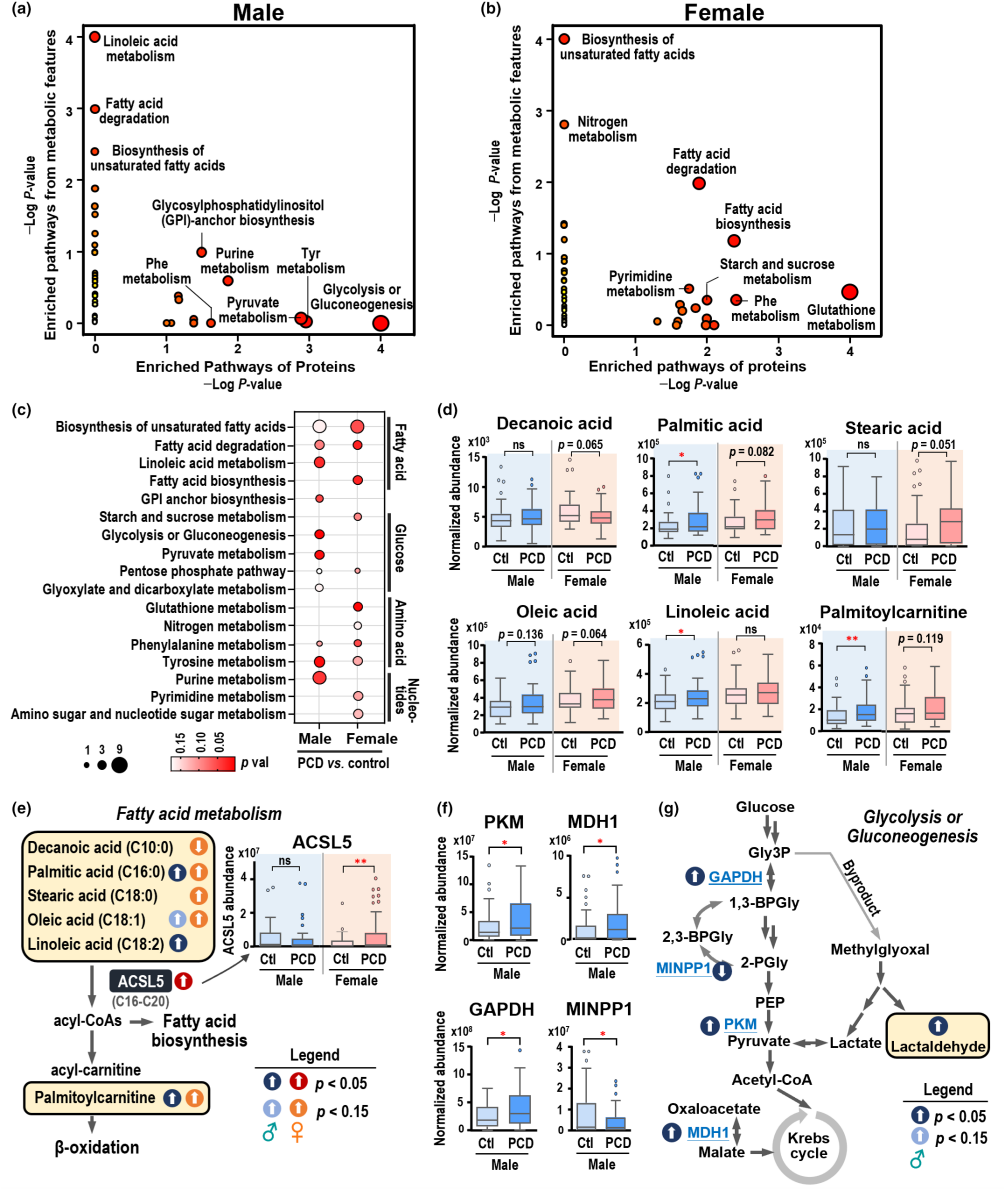
Integration of proteomics and metabolomics revealed alterations in fatty acid metabolism and glycolytic metabolism in PCDS compared to healthy controls. (a, b) Joint‐pathway (multi‐omics) analysis using proteomics and metabolomics data performed by MetaboAnalyst 5.0 in males (a) and females (b). All enrichment results can be found in Table S2. (c) Bubble plots of each individual pathway indicated in (a) and (b) for male‐ and female‐specific comparisons (x‐axis). The enriched metabolic pathways related to PCDS are grouped into different categories including fatty acids, amino acids, glucose and nucleotide metabolism. The size of the bubbles shows the number of biomolecules (proteins or metabolites) involved, and the color denotes the level of significance by *p* value. (d) Box plots of six differentially regulated metabolites involved in fatty acid metabolism in PCDS relative to control. Median values are presented by horizontal lines and the box defines the 25th and 75th percentile of the data. (e) Schematic representation of significantly altered/affected proteins/metabolites in fatty acid degradation and biosynthesis pathways. Arrow denotes regulation trend (upregulation or downregulation) and light/dark color represents statistical significance. (f) The plasma levels of MDH1 and glycolytic enzymes elevated in male subjects with PCDS compared with controls. (g) Graphical overview of glycolysis and TCA cycle with affected enzymes and metabolites highlighted. Ns, not significant. *p < 0.05; **p < 0.01.

The sex‐stratified proteo–metabolite joint pathway analysis on differential proteins and metabolites revealed altered pathways including glutathione metabolism, fatty acid degradation, glycolysis/gluconeogenesis, and tyrosine metabolism, as shown in Figure 3a,b. Detailed protein and metabolite changes within these pathways (*p* < 0.1) are provided in Table S3. Marked disruptions in fatty acid metabolism, that is, biosynthesis of unsaturated fatty acids, linoleic acid metabolism, and fatty acid degradation, were consistent with the metabolomics finding in both sexes. Additionally, glutathione metabolism, phenylalanine metabolism, and pyrimidine metabolism were specifically enriched in women with PCDS. In contrast, glycolysis/gluconeogenesis, pyruvate metabolism, tyrosine metabolism, and purine metabolism were modulated in men with PCDS (Figure 3c). Metabolites involved in fatty acid metabolism, including five fatty acids (decanoic acid, palmitic acid, stearic acid, oleic acid, and linoleic acid) and palmitoylcarnitine, were upregulated in PCDS except for decanoic acid (Figure 3d). ACSL5, activating free long‐chain fatty acids (C16–C20) to produce fatty acyl‐CoA as substrates for lipid synthesis or β‐oxidation, was increased in women but not men with PCDS (Figure 3e). For central carbon metabolism, elevated glycolytic enzymes pyruvate kinase M (PKM), malate dehydrogenase 1 (MDH1), and glyceraldehyde‐3‐phosphate dehydrogenase (GAPDH) alongside decreased multiple inositol polyphosphate phosphatase 1 (MINPP1) indicated altered glucose metabolism at the protein level in male PCDS (Figure 3f). Additionally, levels of lactaldehyde, an intermediate in the methylglyoxal metabolic pathway, were elevated in people with PCDS (Figure 3g).

### 2.5 Disrupted glutathione and amino acid metabolism in PCDS

**FIGURE 4:**
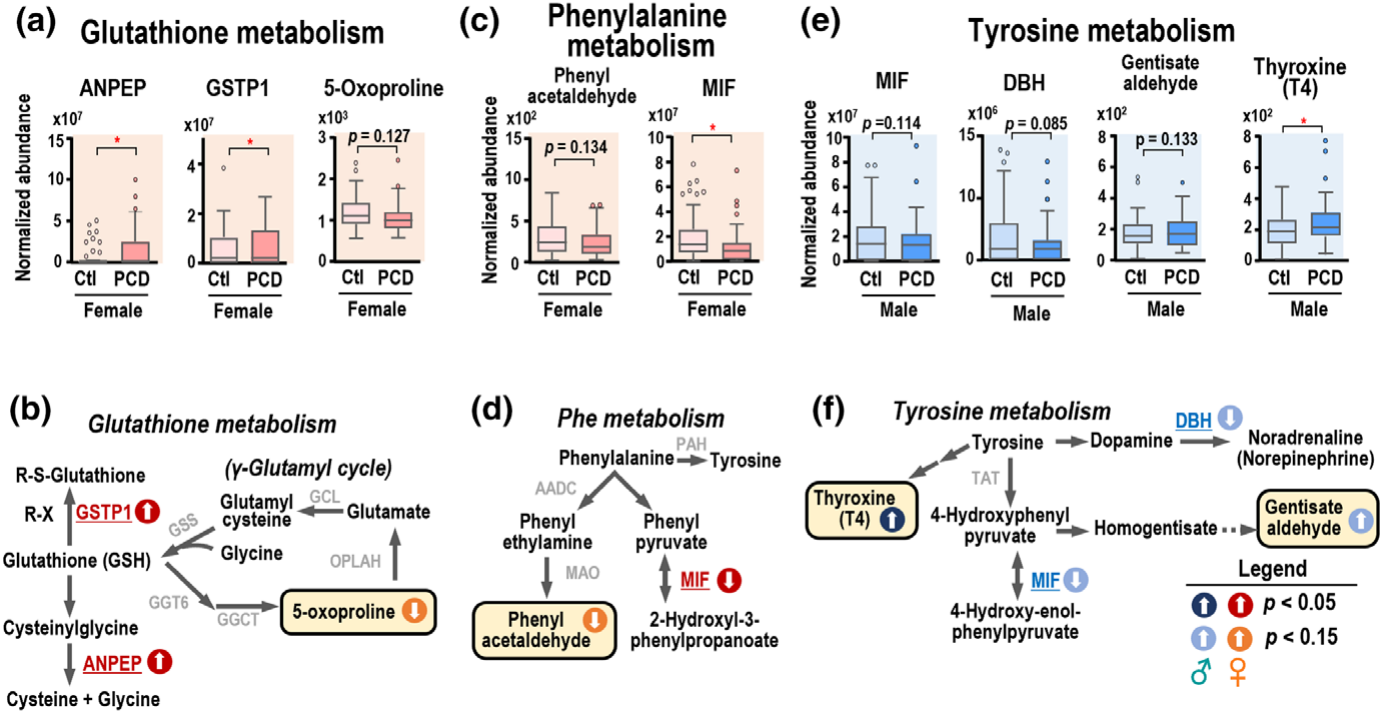
Integrative proteomics and metabolomics approach highlight dysregulated glutathione and amino acid metabolism pathways underlying PCDS. (a) Changes in glutathione metabolism‐related proteins and metabolite in female subjects with PCDS. (b) A representation of molecules involved in glutathione synthesis, degradation and recycling. (c) Reductions in MIF and phenylacetaldehyde, both are associated with phenylalanine metabolism, in female PCDS. (d) Schematic representation of phenylalanine metabolism routes in addition to tyrosine conversion. (e) Changes in tyrosine metabolism‐related proteins and metabolites in male subjects with PCDS. (f) Schematic representation of tyrosine metabolism pathways, including synthesis of dopamine, formation of thyroxine and homogentisic acid. *p < 0.05; **p < 0.01.

In the glutathione pathway, the PCDS group showed a significant increase of aminopeptidase N (ANPEP) and glutathione S‐transferase pi (GSTP1), accompanied by a decreasing trend of pyroglutamic acid (5‐oxoproline) (Figure 4a), indicating the dysregulation of glutathione homoeostasis. ANPEP, with its broad substrate specificity, can remove terminal amino acids from proteins and peptides and has been demonstrated to hydrolyze cysteinylglycine into cysteine and glycine (Uehara et al., 2009), which serve as substrates for glutathione biosynthesis. A schematic overview of the glutathione pathway is summarized in Figure 4b. Phenylalanine metabolism was also affected in women with PCDS, with decreased macrophage migration inhibitory factor (MIF) and phenylalanine derivative phenylacetaldehyde (Figure 4c,d). In men, PCDS showed dysregulated tyrosine metabolism, including altered MIF, dopamine beta hydroxylase (DBH), gentisate aldehyde, and thyroxine (Figure 4e). MIF possesses phenylpyruvate tautomerase activity (Rosengren et al., 1997) utilizing substrates like phenylpyruvate and 4‐hydroxyphenylpyruvate in both phenylalanine and tyrosine metabolism (Figure 4d,f). MIF decreased consistently in male and female PCDS. Tyrosine, precursor of thyroxine (T4) and norepinephrine, is synthesized from phenylalanine by phenylalanine hydroxylase. Increased T4 and decreased norepinephrine‐synthesizing DBH may serve as male‐specific PCDS biomarkers (Figure 4f). Collectively, these data reveal sex‐specific effects of PCDS on glutathione and amino acid metabolism, which may link to oxidative stress and increased thyroxine production.

## 3 DISCUSSION

This study represents the first investigation into the circulating proteo‐metabolomic profiles of PCDS, examining the characteristics and underlying molecular alterations associated with PCDS through a propensity score‐matched sample. Pathway enrichment analysis implicated immune‐inflammatory pathways as significant contributors to PCDS pathology in both sexes. Sex‐specific associations between PCDS and potential biomarkers were identified in this study, with an intriguing observation that men with PCDS showed an enrichment in the pathways of alcohol and cholesterol metabolism. Employing an integrated proteo‐metabolomic approach, we achieved a more comprehensive understanding of the molecular alterations associated with PCDS, thereby offering valuable insights into complex biological processes towards healthy aging. The joint pathway analysis of differential proteins and metabolites revealed disruptions in essential pathways including central carbon metabolism, glutathione metabolism, fatty acid degradation, glycolysis/gluconeogenesis, and tyrosine metabolism, shedding light on the intricate molecular landscape associated with PCDS. Notably, ANPEP, PKM, and MIF have emerged as potential biomarkers, emphasizing their importance in delineating the muscle–brain crosstalk.

In this study, multiple fatty acids (palmitic, stearic, oleic, and linoleic acids) were elevated in participants with PCDS (Figure 3). Fatty acids, which are crucial for brain health, and the significant roles that skeletal muscle plays in fatty acid metabolism are influenced by the processes of ageing and sarcopenia (Al Saedi et al., 2022). The observed elevation in specific fatty acids may be indicative of an age‐related redistribution of body fat. A noteworthy finding in our study was the absence of evidence indicating overall obesity or altered serum lipid profiles in individuals with PCDS (Table S4). Specifically, we observed no significant differences in markers of obesity such as waist circumference, body fat percentage, and body mass index (BMI), nor in serum lipid parameters including total cholesterol, high‐density lipoprotein (HDL), low‐density lipoprotein (LDL), and triglyceride levels. The observed alterations in circulating fatty acids may be attributable to changes in muscle lipid content, a hypothesis that warrants further investigation. Moreover, an increase in circulating fatty acids, exhibiting sex differences, has been reported in the aging process (Darst et al., 2019), and polyunsaturated fatty acids (PUFAs) were related to the muscle hypertrophy (Huang et al., 2023). The elevated levels of palmitoylcarnitine, an intermediate involved in the transport of fatty acids for mitochondrial β‐oxidation, identified in this study, suggest a potential role of mitochondrial dysfunction in PCDS. Our data support the hypothesis that maintaining mitochondrial energetic is crucial for healthy aging. This phenomenon may be a common occurrence in various conditions associated with aging. While previous research has suggested an association between decreased levels of carnitine species and conditions such as frailty or sarcopenia (Kameda et al., 2021; Rattray et al., 2019), this study presents a contrasting pattern. This could potentially indicate a different biological mechanism involving the muscle–brain crosstalk rather than just the skeletal muscle alone, in the context of frailty or sarcopenia.

Men with PCDS exhibited increased glycolytic enzymes PKM, GAPDH, and MDH1 alongside decreased MINPP1 (Figure 3g). Pyruvate kinase catalyzes one of the rate‐limiting steps of glycolysis, and pyruvate kinase muscle isozymes (M2‐type) have been reported to be associated with stroke, neuroinflammation, and cancers. Circulatory PKM2 has been suggested as a potential biomarker for coronary disease and sepsis (Wang et al., 2023; Zhao et al., 2024) and was associated with cognitive function in people with cerebral small vessel disease (Bian et al., 2023). Importantly, our prior research has unequivocally demonstrated the risk associated with cerebral small vessel disease in PCDS (Chung et al., 2022). PKM2, proposed as a novel myokine, is believed to cross the blood–brain barrier and stimulate axonal growth in mice with chronic spinal cord injury, serving as a biomarker of upstream neurological damages (Kodani et al., 2019). In the context of muscle‐brain crosstalk, it is possible that PKM2 is released to counterbalance the neurodegeneration related to PCDS. The enzyme, MINPP1, regulates the catalyzation from 2, 3‐BPG to 2‐PG, participates in glycolytic bypass, and inhibits cancer cell growth in hepatitis B virus‐related hepatocellular carcinoma (Chen et al., 2021). Nonetheless, the elevation of MDH1 in the cerebrospinal fluid of individuals with neurodegenerative disorders has been considered as a generic marker of mitochondrial dysfunction (Heywood et al., 2015). In this study, we hypothesized that the observed augmentation in the levels of MDH1 and reductions in the levels of MINPP1 might also be universal indicators associated with aging, but not uniquely linked to PCDS. Our prior epidemiological research highlighted a distinct sex‐specific feature in the reversibility of PCDS, where men had a higher incidence rate and women, especially those with better nutrition, had a more favorable chance of successful recovery to a healthy state (Lee et al., 2022), a finding that is consistent with existing literature suggesting sexual differences in frailty and cognitive decline (Ruan et al., 2017). Utilizing the proteo‐metabolomic approach, our study offered a comprehensive examination of the impact of sex on the biological processes contributing to PCDS diversity, with specific findings showing a significant distinction between PCDS and control groups in our multivariate OPLS‐DA models (Figure 1b,c). Our pathway analysis underscored distinct sex‐specific effects, notably that women showed a more pronounced disruption in glutathione metabolism (Figure S2h), a process associated with cognitive function at both central and serum levels (Chen et al., 2022). This observation aligns with prior research suggesting sex‐specific differences in glutathione antioxidant defenses that could contribute to sex disparities in age‐related diseases (Wang et al., 2020). Our findings of elevated ANPEP and GSTP1, along with reduced pyroglutamic acid in PCDS (Figure 4), indicate a disturbance in glutathione homeostasis. As the primary intracellular antioxidant, glutathione safeguards against oxidative damage, and its depletion can lead to an increase in reactive oxygen species and exacerbated inflammation, with the intermediate metabolite pyroglutamic acid, which is connected to glutathione turnover via the γ‐glutamyl cycle, showing decreased levels in senescent myoblasts in cell models (Mohd Sahardi et al., 2023). Therefore, it can be postulated that the diminished systemic pyroglutamic acid levels might signal muscle aging, despite the decrease in plasma not being significant. ANPEP has been recognized as the type II transmembrane protein CD13. The soluble form of ANPEP (sANPEP) can be secreted from astrocytes and positively regulates microglial activation and neuroinflammation (Kim et al., 2022). It may suggest a state of neuroinflammation associated with PCDS, as indicated by the heightened circulatory ANPEP levels identified in plasma in this study.

Macrophage migration inhibitory factor (MIF) levels were decreased in both men and women with PCDS compared to controls (*p* < 0.05 in women, *p* = 0.11 in men, Figure 4c,e). MIF, functioning as a phenylpyruvate keto‐enol tautomerase and participating in tyrosine and phenylalanine metabolism, has its enzymatic activity's contribution to physiological and pathological roles still under debate. One study showed its tautomerase activity is dispensable for its growth‐regulatory properties (Fingerle‐Rowson et al., 2009), while another study demonstrated tautomerase activity‐deficient MIF significantly alleviated high‐fat diet‐induced obesity and adipose tissue inflammation (Li et al., 2020). Beyond its enzymatic activity, MIF is known as a versatile cytokine implicated in inflammation and associated conditions such as obesity, diabetes mellitus, autoimmune diseases, and cancer (Grieb et al., 2010). A prior study established a biomarker risk score utilizing circulating MIF levels and five additional proteins to prognosticate sarcopenia in patients with chronic heart failure and chronic obstructive pulmonary disease that highlights the potential role of MIF in certain myopathies (Qaisar et al., 2021). Despite some studies noting elevated MIF levels in certain inflammatory myopathies, we found reduced MIF in PCDS participants, and considering that PCDS could signify early‐stage accelerated aging phenotypes before sarcopenia and dementia, higher MIF levels could potentially suggest progressive muscle loss and inflammation at the later stage.

In men with PCDS, tyrosine metabolism, a pathway that influences the production of catecholamine and thyroid hormones, is altered, evidenced by increased thyroxine levels and decreased dopamine‐β‐hydroxylase (DBH) levels, a protein primarily expressed in the central nervous system and found to have reduced activity in post‐mortem hippocampus and neocortex samples from Alzheimer's disease patients (Mustapic et al., 2013). In addition, there is a reduction in genotype‐independent plasma DBH activity in Alzheimer's disease, particularly in the early stage, compared to levels observed in the late stages of the disease and healthy individuals (Mustapic et al., 2013). The observed reduction in DBH levels in PCDS may be due to a compensatory mechanism addressing the loss of noradrenergic neurons, while the serum thyroxine levels, controlled by the hypothalamic‐pituitary‐thyroid (HPT) axis, are influenced by factors such as nutritional status, illness, and aging (Fliers et al., 2015). Increased free thyroxine (T4) has been reported in frail individuals compared to robust and pre‐frail ones (Sargent et al., 2023), which is in line with our observations. While both increased and decreased T4 serum levels have been observed in sarcopenia and myopathies (Bloise et al., 2018), reflecting potential variations during the disease course, it remains unclear whether these alterations in thyroid hormone status are a cause or consequence of frailty or PCDS.

To investigate the potential tissue sources of the PCDS biomarkers, mRNA expression levels in brain and muscle were procured from the publicly accessible Human Protein Atlas database, as encapsulated in Figure S3. We noted that most proteins were not exclusively expressed in either muscle or brain, except for ANPEP (mostly in smooth muscle) and DBH (mainly in the brain). In particular, ACSL5, ANPEP, and MDH1 were highly expressed in muscle compared to brain subregions, while MIF and DBH were highly expressed in brain. Other proteins (GAPDH, GSTP1, MINPP1, and PKM) were detected simultaneously in the brain and muscles. Moreover, almost all PCDS‐related proteins and metabolites have been quantified in circulatory forms (Figure S3b,c). Concerning ANPEP, GAPDH, GSTP1, PKM, and MIF were identified in the secretome of human skeletal muscle cells (Florin et al., 2020), these alterations in circulation were mediated by skeletal muscle, to the certain extent. For fatty acids, the ability to cross the BBB via simple diffusion or various fatty acid transport proteins has been proposed (Mitchell et al., 2011). Whether the increase in the plasma fatty acids (decanoic acid, palmitic acid, stearic acid, oleic acid, and linoleic acid) is facilitated by increased release from skeletal muscle and contributes to the cognitive decline warrant further investigation.

Despite all the efforts ran into the study, there are still some limitations. First, the detected serum proteins are relatively abundant, which limits our protein biomarker identification from incorporating myokines or growth factors. We acknowledge that our analysis was limited to a subset of detectable proteins. It is possible that other undetected proteins also contribute significantly to the pathogenesis of PCDS. Second, the cross‐sectional study design limits the exploration of causal relationships. However, through meticulously designed and well‐matched pairs, the process may be more efficient and dependable in pinpointing potential biomarkers, whilst ILAS, being a longitudinal cohort study, provides us the prospect to scrutinize relationships utilizing repeated measures as additional data becomes accessible. Last but not least, while PCDS, a combined phenotype of cognitive and mobility decline, prompts interest in whether the observed PCDS‐related alterations are due to muscle or brain, the design of this propensity score matching case–control study precludes comparison between those with only mobility or cognitive impairment, in addition to controls and PCDS. Despite its limitations, this study's robustness, as a trailblazing investigation into PCDS biomarkers—a reversible phenotype of accelerated aging leading to disability, dementia, and mortality—not only paves the way for potential early and simple PCDS diagnosis but also enables the assessment of the prognosis of PCDS interventions.

In conclusion, utilizing the untargeted proteo‐metabolomic approach has led to the discovery of novel proteo‐metabolite biomarkers linked with PCDS, encompassing numerous energy metabolism and glutathione pathways. Notably, the plasma proteins ANPEP, PKM, and MIF could be assembled as a panel of biomarkers for PCDS, paving the way for clinical applications and the exploration of the precise biomechanisms of PCDS or healthy aging. These findings shed light on the intricate muscle‐to‐brain interrelationships and offer insightful perspectives on the pathophysiology of PCDS, marking a significant stride forward on the journey to healthy longevity.

## 4 EXPERIMENTAL PROCEDURES

### 4.1 Study design and population

The I‐Lan Longitudinal Aging Study (ILAS) investigated the interrelationships among frailty, sarcopenia, and cognitive decline throughout aging. Detailed information on the ILAS study design, recruitment procedure, and data collection has been reported in previous studies (Lee et al., 2022; Liu et al., 2020). In brief, the ILAS study recruited community‐dwelling adults ≥50 years. Individuals unable to effectively communicate with research nurses, with a limited life expectancy due to significant illnesses, those presently residing in long‐term facilities, and individuals incapable of completing assessments due to existing disabilities were excluded from the studies. Data from 855 participants who completed the omics analysis were enrolled for analysis. To eliminate the impact due to major known confounding bias caused by the demographic and clinical characteristics of participants and improve the stability of our findings, a propensity score matching (PSM)‐designed case–control approach was applied (Seeger et al., 2005). PCDS is defined as the simultaneous presence of mobility impairment without disability (MIND: slow gait or/and weak handgrip) and cognitive impairment without dementia (CIND: ≥1.5 SD below the mean for age‐, sex‐, and education‐matched norms in any cognitive domain but without dementia) (Chung et al., 2021; Lee et al., 2022). Comprehensive neuropsychological assessments were conducted to evaluate multiple cognitive domains in this study. These domains included: (1) verbal memory, assessed using delayed recall in the Chinese Version Verbal Learning Test (CVVLT); (2) language, evaluated through the Boston Naming Test (BNT) and the categorical (animal) Verbal Fluency Test (VFT); (3) visuospatial function, measured by the Taylor Complex Figure Test (TCF); and (4) executive function, assessed using the Backwards Digit Test (BDT) and the Clock Drawing Test (CDT). Finally, we successfully paired 230 participants with case (PCDS) and control (robust) (Figure 1a). All participants provided written informed consent document through a process facilitated by research nurses. The study protocol obtained ethical approval from the institutional review boards of National Yang‐Ming University (YM103008). The study design and executive procedures adhered to the principles in accordance with the Declaration of Helsinki.

### 4.2 Proteomics analysis

Fasting peripheral blood was drawn using EDTA‐coated vacuum tubes. After centrifugation at 4°C and 3000 ***g*** for 10 min, the plasma samples were obtained and stored at −80°C until analysis. Plasma samples were processed for proteomic analysis using the SMART Digest Trypsin kit (Thermo Fisher Scientific). Briefly, 10 μL of each plasma sample was diluted, supplemented with digestion buffer, and incubated at 70°C for 2 h to achieve protein tryptic digestion. The resulting peptides were reduced, iodoacetamide‐alkylated, and purified via solid‐phase extraction on SOLAμ plates. Prior to nanoLC‐nanoESI‐MS/MS analysis on an Orbitrap Elite mass spectrometer, the samples were desalted using C18 ZipTip (Merck). A segmented gradient elution over 210 min was utilized to separate peptides on a C18 column. Data were acquired in a data‐dependent mode with dynamic exclusion of 60s. RAW data were searched against the human Uniprot database using Proteome Discoverer version 2.4 (Thermo Fisher Scientific). Database search was performed with Mascot and Sequest HT allowing up to one missed cleavage, cysteine carbamidomethylation as static modification, and methionine oxidation as variable modification. Precursor and fragment ion tolerances were set to 20 ppm and 0.8 Da, respectively. Normalization was performed using the sum of all peptide amounts, while protein abundances at MS1 level were quantified by summing abundances of their associated unique peptides. Proteins not detected in at least 50% samples were excluded, and about 500 proteins were retained after the filtering.

### 4.3 Metabolomics analysis

Plasma samples (40 μL) were spiked with internal standards (1 ppm lysine‐^13^C_6_, 1 ppm stearic acid‐^13^C_18_) before deproteinization using 160 μL 100% methanol. After centrifugation and lyophilization, the supernatants were reconstituted in water for LC–MS analysis on a Waters Xevo G2‐S QTof tandem mass spectrometer coupled to an Acquity UPLC with a BEH C18 column (2.1 × 100 mm 1.7 μm, Waters). A 9‐min gradient elution method was utilized with 1%–100% mobile phase B (acetonitrile containing 0.1% ammonium hydroxide) over 4 min, followed by a return to initial conditions. Mass calibration was performed continuously using leucine enkephalin. MS^E^ data were acquired in negative mode from 50 to 1200 m/z under low and high collision energy. A pooled quality control (pQC) sample was prepared by mixing aliquots from 60 plasma samples and analyzed repeatedly to normalize chemical feature abundances across analysis batches. Chemical features were represented by retention time, m/z, and ion intensity by Progenesis QI (Nonlinear Dynamics) software. Subsequently, ion intensities were normalized against the pQC reference to reduce batch effects. Triplicate measurements of each individual sample were then averaged before statistical analysis.

### 4.4 Pathway analysis

Kyoto Encyclopedia of Genes and Genomes (KEGG) pathway analyses were performed for the proteins differentially expressed in PCDS by DAVID Bioinformatics Resources 6.8 (http://david.abcc.ncifcrf.gov/). Two modules in MetaboAnalyst (https://www.metaboanalyst.ca/) were used for pathway enrichment analysis: (i) Metabolite set enrichment analysis for metabolomics data; and (ii) Joint‐Pathway analysis for integration of proteomics and metabolomics data. Among the metabolites annotated by MetaboAnalyst, the peaks corresponding to fatty acids were further confirmed by comparing their retention times and m/z values with those of authentic standards.

### 4.5 Statistical analysis

Numerical variables are presented as the mean plus/minus standard deviation, while categorical variables are expressed as numbers (proportions). Descriptive characteristics were compared using student's *t* test, one‐way ANOVA, or chi‐square analysis as appropriate. A relaxed threshold for cut‐off *p*‐value was used (*p* < 0.15) to filter potential differential expression of omics data. The orthogonal partial least‐squares discriminant analysis (OPLS‐DA) among the selected groups was obtained by MetaboAnalyst 5.0. Bar charts and bubble plots were conducted using GraphPad Prism v9.0. Baseline variables, including age, sex, smoking, drinking, education years, and chronic conditions (i.e., hypertension, congestive heart failure, stroke, diabetes, chronic obstructive pulmonary disease, and chronic kidney disease) were included in a multivariable logistic model for the propensity score. The nearest neighbor algorithm was used in the matching process at a ratio of 1:1 to generate 115 pairs. A two‐sided *p*‐value of <0.05 was considered statistically significant. All analyses were conducted using the SAS statistical package, version 9.4 (SAS Institute, Inc., Cary, NC, USA).

## AUTHOR CONTRIBUTIONS

Y.‐L.H. and W.‐J.L. wrote the manuscript. W.‐J.L. collected the phenotype data and coordinated the writing process. W.‐J.C. and Y.‐L.H. analyzed data and drafted figures. C.‐H.H. contributed to the acquisition of LC–MS data. C.‐H.L. evaluated the quality of the data included. L.‐N. P., C.‐P. C., and L.K.‐C. contributed to the study design and discussion. L.K.‐C. supervised the project. All authors contributed to the article and approved the submitted version.

## CONFLICT OF INTEREST STATEMENT

The authors declare no competing interests.

## Supporting information


Appendix S1.

## Data Availability

The data supporting the findings of this study are available from the corresponding author upon reasonable request.
