# Supplementary material for: Proteo‐metabolomic insights for early dual physical and cognitive impairments: A search for biomarkers of healthy aging based on muscle–brain crosstalk
Source: Aging Cell. 2024 Nov 8;24(3):e14407. doi: 10.1111/acel.14407 (PMC11896308; doi:10.1111/acel.14407)
Supplement: Supplementary file 1 — Appendix S1. [file ACEL-24-e14407-s001.pdf]

Supporting Information

Proteo-metabolomic insights for early dual physical and cognitive impairments: a search for biomarkers of healthy ageing based on muscle–brain crosstalk

Yi-Long Huang[1], Wei-Ju Chang[1], Chen-Hua Huang[2], Chao-Hsiung Lin[1,2], Li-Ning Peng[1,3], Chih-Ping Chung[1,4] , Liang-Kung Chen[1,3,5] and Wei-Ju Lee[1,6,7,*]

[1]Center for Healthy Longevity and Aging Sciences, National Yang Ming Chiao Tung University, Hsinchu, Taiwan; [2]Department of Life Sciences and Institute of Genome Sciences, National Yang Ming Chiao Tung University, Hsinchu, Taiwan; [3]Center for Geriatrics and Gerontology, Taipei Veterans General Hospital, Taipei, Taiwan; [4]Department of Neurology, Neurological Institute, Taipei Veterans General Hospital, Taipei, Taiwan; [5]Taipei Municipal Gan-Dau Hospital (Managed by Taipei Veterans General Hospital), Taipei, Taiwan; [6]Department of Geriatric Medicine, School of Medicine, National Yang Ming Chiao Tung University, Hsinchu, Taiwan; [7]Department of Family Medicine, Taipei Veterans General Hospital Yuanshan Branch, Yilan, Taiwan

Figure S1 Untargeted metabolomics data processing evaluation.

Figure S2 Metabolic features and pathways significantly altered in PCDS based on sex-combined analyses.

Figure S3 Tissue-specific expression profiles of proteins and metabolites associated with PCDS in brain, muscle and blood.

Table S1 Participant characteristics matched by propensity scores, stratified by physio-cognitive decline syndrome and sex.

Table S2 PCDS-related proteins with consistent regulation trends in previous frailty studies.

Table S3 The protein-metabolite joint pathway analysis to identify pathways associated with PCDS in both sexes.

Table S4 Body adiposity measurements and lipid profile stratified by physio-cognitive decline syndrome

Figure S1

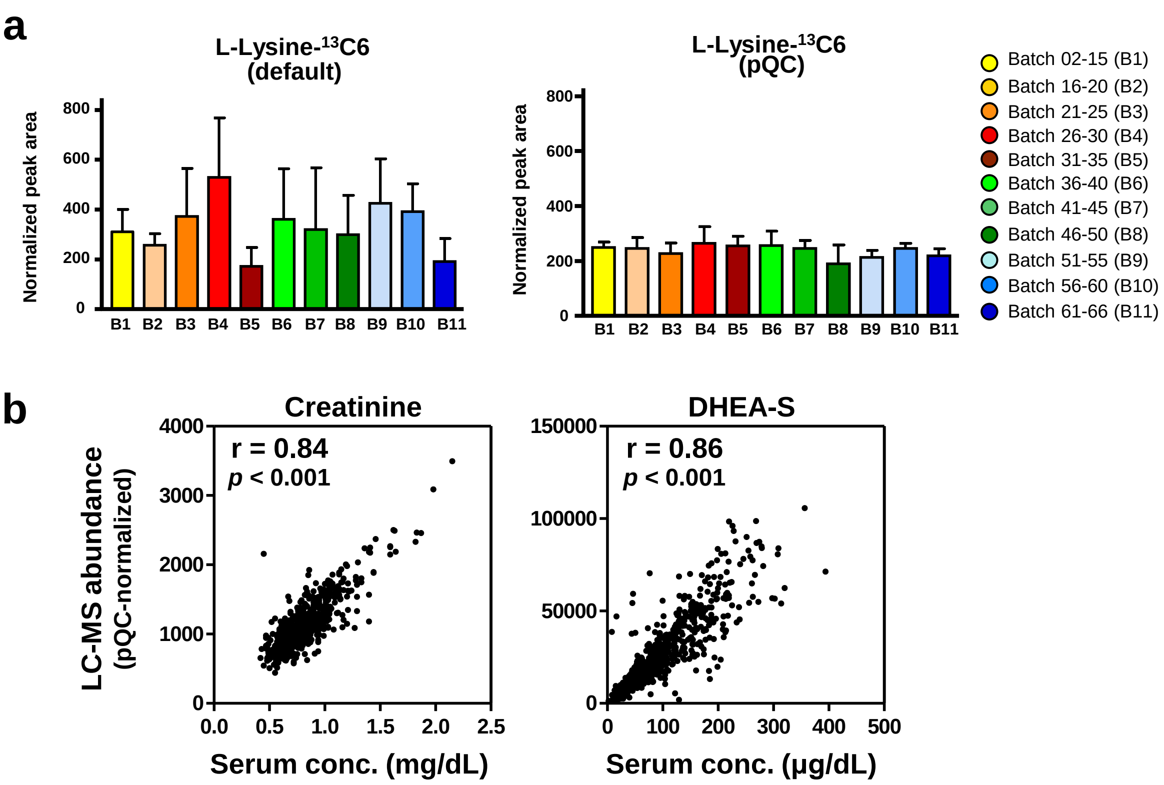

Figure S1. Untargeted metabolomics data processing evaluation. (a) Levels of internal standard (L-lysine-$^{13}C_6$) spiked with defined concentration in various batches upon default normalization and pQC normalization. **(b)** Comparison of DHEA-S and creatinine concentrations as determined in plasma by clinical chemical analyzer and pQC-normalized abundance by MS in ILAS1 participants. The Pearson correlation coefficient r was used to evaluate the linear relationship**.** pQC, pooled quality control.

Figure S2

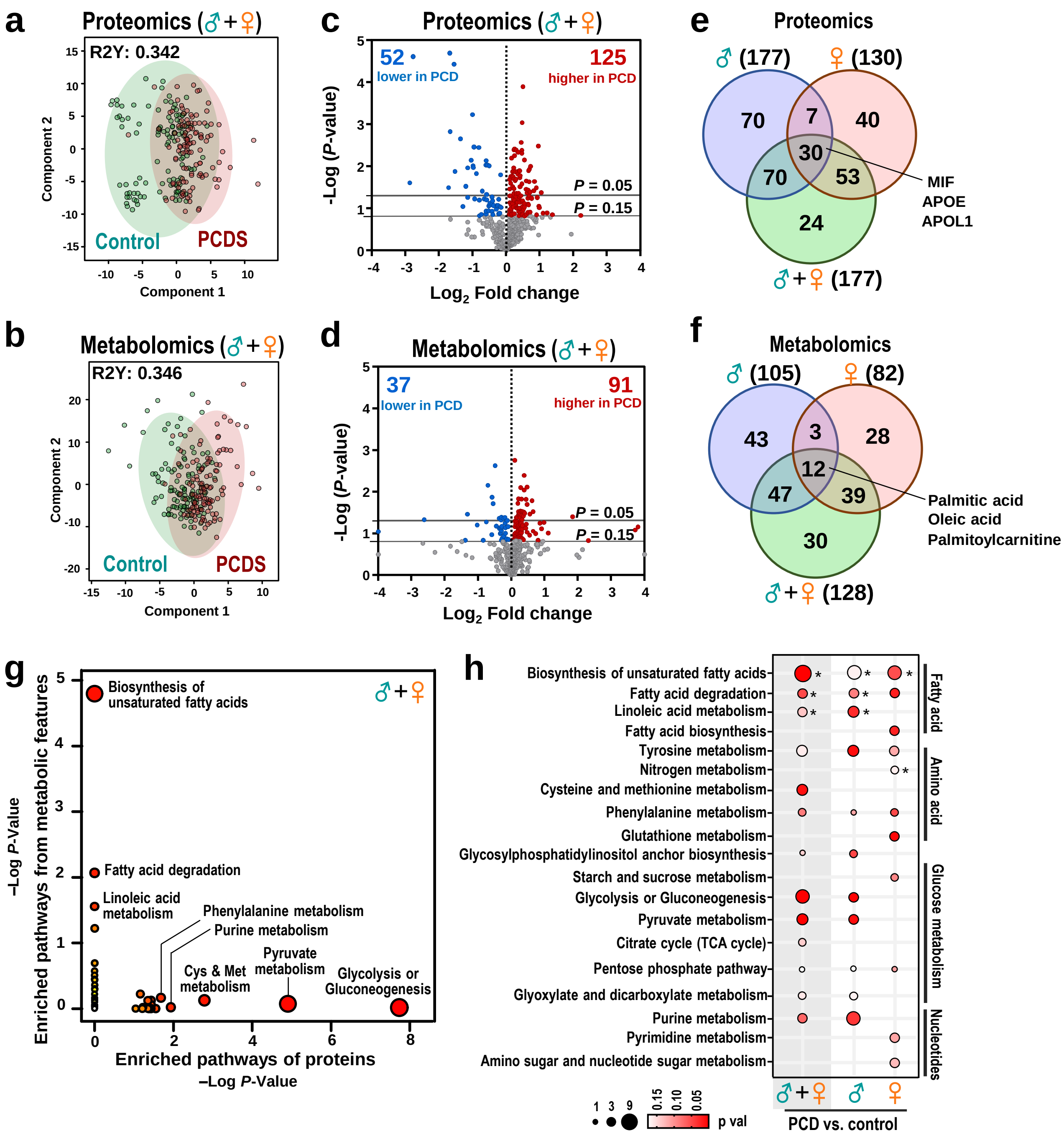

Figure S2. Proteomic and metabolomic associations for PCDS based on sex-combined analyses. (a,b) OPLSDA models of proteomic profile (a) and untargeted metabolomic profile (a) comparing PCDS and control in sex-combined analysis. **(c,d)** Volcano plots showing the differential proteins (c) and metabolic features (d) between PCDS and controls when both sexes were analyzed together. Limma model, implemented in MetaboAnalyst, was used to perform differential analysis using sex as a covariate **(e,f)** Venn diagrams representing common and unique proteins (e) and metabolic features (f) associated with PCDS from the female-specific (red), male-specific (blue), sex-combined (green) analyses. **(g)** Joint pathway analysis using combined-sex proteomic and metabolomic profile for PCDS by MetaboAnalyst 5.0. **(h)** Bubble plot of each individual pathway indicated in (h) for sex-combined analysis. See Fig. 3 for male- and female-specific comparisons. * denotes $P_{(Metabolomics)} < 0.05$; other pathways listed were $P_{(Proteomics)} <0.05$.

Figure S3

a

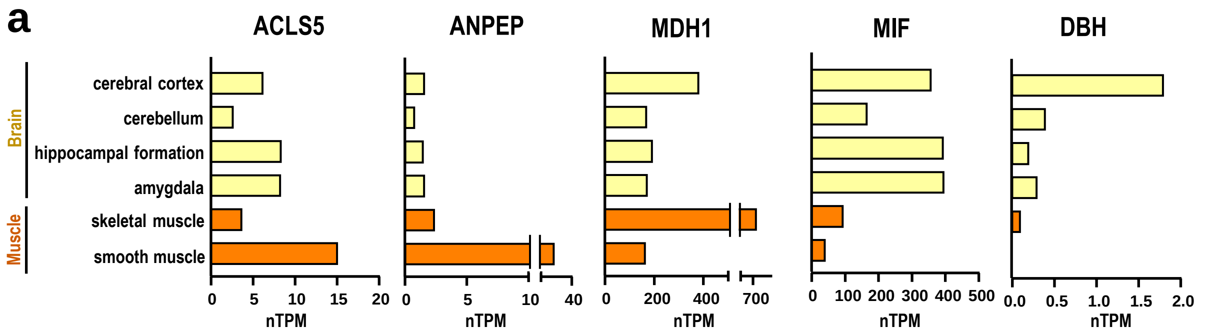

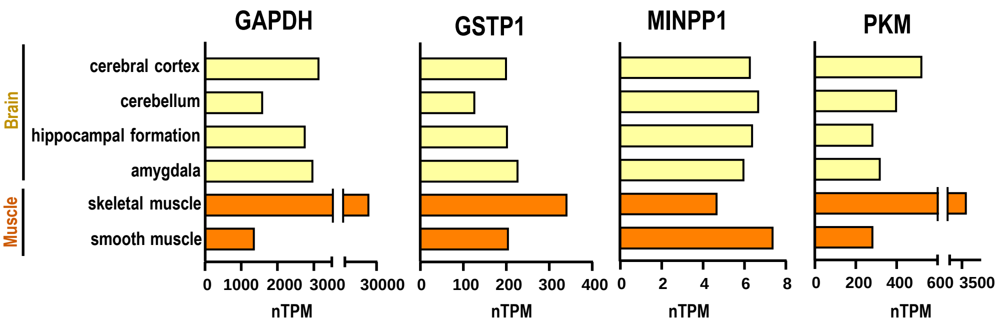

b

Protein	Plasma conc.	
ACSL5	NA	-
ANPEP	1.2	mg/L
DBH	1.6	mg/L
GAPDH	1.4	mg/L
GSTP1	69	µg/L
MDH1	49	µg/L
MIF	5.7	µg/L
MINPP1	370	µg/L
PKM	310	µg/L

c

Tissue locations	Palmitic acid	Palmitoyl carnitine	Stearic acid	Decanoic acid	Oleic acid	Linoleic acid	Pyroglutamic acid	Thyroxine (T4)
Brain			+				+	
Skeletal muscle	+		+		+	+		+
Blood	122 ± 48 µM	1.9 (1.01-2.23) µM	48.8 ± 21 µM	11.0 ± 6 µM	122 ± 56 µM	83.8 ± 38 µM	19.5 ± 3.7 µM	0.016 (0.0064-0.064) nM

Not available: phenylacetaldehyde, gentisate aldehyde

Figure S3. Tissue-specific expression profiles of proteins and metabolites associated with PCDS in brain, muscle and blood. (a) The mRNA levels (based on TPM values) of the PCDS-related genes across different brain and muscle tissues in the Consensus datasets of the Human Protein Atlas (https://www.proteinatlas.org/) resource. **(b)** Circulating blood concentrations PCDS-related proteins in the Human Protein Atlas database. **(c)** PCDS-related metabolites with blood concentration or tissue location data in HMDB database (https://hmdb.ca/). TPM, transcripts per million.

Table S1

Table S1. Participant characteristics matched by propensity scores, stratified by physio-cognitive decline syndrome and gender.

	All				Male			Female		
	Total (n=230)	Control (n=115)	PCDS (n=115)	*p*	Control (n=59)	PCDS (n=59)	*p*	Control (n=56)	PCDS (n=56)	*p*
**Age (years)**	67.0 ± 8.9	66.7 ± 8.5	67.2 ± 9.3	0.658	68.5 ± 8.9	67.0 ± 9.3	0.370	64.8 ± 7.7	67.4 ± 9.4	0.104
**Sex (female)**	112 (48.7)	56 (48.7)	56 (48.7)	1.000	0 (0.0)	0 (0.0)	1.000	56 (100.0)	56 (100.0)	1.000
**Post-menopause**	-	-	-	-	-	-	-	54 (96.4)	55 (98.2)	0.558
**Education (years)**	5.0 ± 4.8	5.2 ± 5.0	4.8 ± 4.7	0.522	5.7 ± 4.9	5.4 ± 4.5	0.725	4.7 ± 5.0	4.2 ± 4.8	0.580
**Smoke**	35 (15.2)	14 (12.2)	21 (18.3)	0.199	13 (22.0)	21 (35.6)	0.104	1 (1.8)	0 (0.0)	1.000
**Drink**	67 (29.1)	34 (29.6)	33 (28.7)	0.885	22 (37.3)	25 (42.4)	0.573	12 (21.4)	8 (14.3)	0.460
**Hypertension**	97 (42.2)	48 (41.7)	49 (42.6)	0.894	22 (37.3)	23 (39.0)	0.850	26 (46.4)	26 (46.4)	1.000
**CHF**	7 (3.0)	3 (2.6)	4 (3.5)	1.000	2 (3.4)	3 (5.1)	1.000	1 (1.8)	1 (1.8)	1.000
**Stroke**	0 (0.0)	0 (0.0)	0 (0.0)	1.000	0 (0.0)	0 (0.0)	1.000	0 (0.0)	0 (0.0)	1.000
**Diabetes**	40 (17.4)	19 (16.5)	21 (18.3)	0.728	8 (13.6)	11 (18.6)	0.452	11 (19.6)	10 (17.9)	0.809
**COPD**	0 (0.0)	0 (0.0)	0 (0.0)	1.000	0 (0.0)	0 (0.0)	1.000	0 (0.0)	0 (0.0)	1.000
**CKD**	0 (0.0)	0 (0.0)	0 (0.0)	1.000	0 (0.0)	0 (0.0)	1.000	0 (0.0)	0 (0.0)	1.000
**Grip strength (kg)**	26.1 ± 8.7	30.1 ± 7.6	22.0 ± 7.7	**<0.001**	36.1 ± 5.2	27.0 ± 7.1	**<0.001**	23.8 ± 3.6	16.7 ± 3.5	**<0.001**
**Walking speed (m/s)**	1.5 ± 0.5	1.7 ± 0.4	1.4 ± 0.5	**<0.001**	1.8 ± 0.4	1.4 ± 0.5	**<0.001**	1.6 ± 0.4	1.3 ± 0.5	**<0.001**
**CVVLT**	6.1 ± 2.2	6.8 ± 1.6	5.4 ± 2.4	**<0.001**	6.7 ± 1.7	4.9 ± 2.5	**<0.001**	6.9 ± 1.5	6.0 ± 2.3	**0.009**
**BNT**	9.4 ± 3.2	10.5 ± 2.7	8.3 ± 3.2	**<0.001**	11.0 ± 2.6	8.8 ± 3.2	**<0.001**	9.9 ± 2.7	7.9 ± 3.2	**0.001**
**BDT**	3.0 ± 2.0	4.0 ± 1.5	2.1 ± 2.1	**<0.001**	4.2 ± 1.4	2.3 ± 1.9	**<0.001**	3.8 ± 1.6	1.9 ± 2.2	**<0.001**
**VFT**	13.8 ± 4.5	15.4 ± 4.3	12.3 ± 4.2	**<0.001**	16.1 ± 4.0	12.8 ± 4.4	**<0.001**	14.7 ± 4.5	11.7 ± 3.9	**<0.001**
**TCF**	28.3 ± 7.9	30.5 ± 5.9	26.1 ± 9.0	**<0.001**	31.4 ± 5.3	26.3 ± 7.9	**<0.001**	29.7 ± 6.3	25.8 ± 10.2	**0.019**
**CDT**	7.3 ± 2.6	8.0 ± 2.2	6.5 ± 2.8	**<0.001**	8.5 ± 1.7	7.0 ± 2.6	**<0.001**	7.5 ± 2.5	5.9 ± 2.8	**0.002**

Means ± standard deviation (SD) are shown for continuous variables; PCDS, Physio-Cognitive Decline Syndrome; CHF, congestive heart failure; COPD, chronic obstructive pulmonary disease; CKD, chronic kidney disease; CVVLT, Chinese Version Verbal Learning Test; BNT, Boston Naming Test; BDT, Backward Digit Span Test; VFT, Verbal Fluency Test; TCF, Taylor Complex Figure Test; CDT, Clock Drawing Test.

Table S2

Table S2. PCDS-related proteins with consistent regulation trends in previous frailty studies.

Male PCDS (current study)					Frailty (previous study)		
**Acc. no.**	**Gene**	**Protein**	**PCD/CTL**	***P* value**	**Reported trend in frailty**	***P* value**	**Ref**
P01033	TIMP1	Metalloproteinase inhibitor 1	1.738	0.054	Up-regulated	0.026	1
P00441	SOD1	Superoxide dismutase [Cu-Zn]	3.098	0.059	Up-regulated	0.008	1
P01876	IGHA1	Immunoglobulin heavy constant alpha 1	1.216	0.069	Up-regulated	0.012	1
P01034	CST3	Cystatin-C	1.386	0.073	Up-regulated	0.020	1
P06702	S100A9	Protein S100-A9	1.554	0.115	Up-regulated	0.009	1
P08493	MGP	Matrix Gla protein	2.38	0.022	Up-regulated	0.026	2
P00740	F9	Coagulation factor IX	1.19	0.046	Up-regulated	0.0004	2

Female PCDS (current study)					Frailty (previous study)		
**Acc. no.**	**Gene**	**Protein**	**PCD/CTL**	***P* value**	**Reported trend in frailty**	***P* value**	**Ref**
P09211	GSTP1	Glutathione S-transferase pi 1	3.35	0.037	Up-regulated	0.0153	2
P23142	FBLN1	Fibulin 1	0.63	0.039	Down-regulated	6.42E-05	2

1. Landino, et al. Characterization of the plasma proteomic profile of frailty phenotype. Geroscience 43, 1029-1037 (2021). **InCHIANTI cohort (Italy)**
2. Sathyan et al., Plasma proteomic profile of frailty. Aging Cell. 19, e13193 (2020). **LonGenity cohort (USA)**

Table S3

Table S3. The protein-metabolite joint pathway analysis to identify pathways associated with PCDS in each gender.

Female PCDS *vs.* Female Control

No.	pathways	Total	hits_protein	hits_compound	P_cpd	P_protein	P_value	-LogP	Holm p	FDR
1	**Glutathione metabolism**	75	2 (ANPEP ↑, GSTP1↑)	1 (5-Oxoproline↓)	0.5818	**0.0007**	**0.0037**	2.43	0.182	0.182
2	**Fatty acid degradation**	80	1 (ACSL5↑)	2 (Palmitoylcarnitine↑, Palmitic acid↑)	0.0987	**0.0330**	**0.0219**	1.66	1	0.374
3	**Fatty acid biosynthesis**	28	1 (ACSL5↑)	2 (Decanoic acid↓, Palmitic acid↑)	0.2522	**0.0136**	**0.0229**	1.64	1	0.374
4	**Phenylalanine metabolism**	27	1 (MIF↓)	1 (Phenylacetaldehyde↓)	0.6642	**0.0128**	**0.0492**	1.31	1	0.519
5	**Biosynthesis of unsaturated fatty acids**	34	0	6 (Palmitic acid↑, Stearic acid↑, Oleic acid↑, Linoleic acid ↑, Arachidonic acid ↑, Eicosapentaenoic acid↑)	**0.0093**	1	0.0529	1.28	1	0.519
6	**Starch and sucrose metabolism**	49	1 (GPI↓)	1 (D-Glucose↓)	0.6642	0.0270	0.0902	1.04	1	0.567

Male PCDS *vs.* Male Control

No.	pathways	Total	hits_protein	hits_compound	P_cpd	P_protein	P_value	-LogP	Holm p	FDR
1	**Glycolysis or Gluconeogenesis**	68	3 (GAPDH↑, MINPP1↓, PKM↑)	0	1	**9.59E-05**	**0.00098**	3.0074	0.049	**0.049**
2	**Tyrosine metabolism**	78	2 (MIF↓, DBH↓)	2 (Thyroxine↑, Gentisate aldehyde↑)	0.9693	**0.0011**	**0.00825**	2.0836	0.404	0.150
3	**Pyruvate metabolism**	58	2 (PKM↑, MDH1↑)	1 (Lactaldehyde↑)	0.9108	**0.0013**	**0.00898**	2.0466	0.431	0.150
4	**Linoleic acid metabolism**	4	0	4 (Linoleic acid↑, 9(10)-EpOME↓, 12(13)-EpOME↓, 13(S)-HPODE↓)	**0.0043**	1	**0.02763**	1.5586	1	0.345
5	**Purine metabolism**	195	2 (FHIT↑, PKM↑)	4 (CAIR↑, Inosinic acid↑, Xanthosine↓, Inosine 5'-diphosphate↑)	0.4448	**0.0134**	**0.03653**	1.4373	1	0.365
6	**Glycosylphosphatidylinositol (GPI)-anchor biosynthesis**	34	1 (GPLD1↑)	0	0.2585	**0.0316**	**0.04743**	1.3239	1	0.395
7	**Fatty acid degradation**	36	0	3 (Palmitic acid↑, Palmitoylcarnitine↑, omega-Hydroxy fatty acid↑)	0.0169	1	0.08593	1.0658	1	0.614

Table S4

Table S4. Body adiposity measurements and lipid profile stratified by physio-cognitive decline syndrome

	Total (n=230)	Control (n=115)	PCDS (n=115)	*p*
**Body total fat (%)**	30.9±9.3	30.7±9.6	31.0±9.0	0.842
**Waist circumference(cm)**	85.8±9.1	85.6±8.9	86.0±9.3	0.738
**Body mass index (kg/m^2)**	24.9±4.0	25.2±4.2	24.7±3.7	0.361
**Total cholesterol (mg/dL)**	189.8±32.4	192.7±32.2	186.9±32.5	0.172
**Triglyceride (mg/dL)**	119.3±69.8	116.1±55.2	122.5±82.0	0.489
**High density lipoprotein (mg/dL)**	53.2±13.9	53.0±13.1	53.5±14.7	0.799
**Low density lipoprotein (mg/dL)**	113.6±29.6	116.1±29.6	111.1±29.6	0.207
